# QuLinePlus: extending plant breeding strategy and genetic model simulation to cross-pollinated populations—case studies in forage breeding

**DOI:** 10.1038/s41437-018-0156-0

**Published:** 2018-10-27

**Authors:** Valerio Hoyos-Villegas, Vivi N Arief, Wen-Hsi Yang, Mingzhu Sun, Ian H DeLacy, Brent A Barrett, Zulfi Jahufer, Kaye E Basford

**Affiliations:** 1 AgResearch Lincoln Research Centre, PB 4749, Christchurch, New Zealand; 2 School of Agriculture and Food Sciences, The University of Queensland, Brisbane, QLD 4072 Australia; 3 School of Mathematics and Physics, Centre for Applications in Natural Resource Mathematics (CARM), The University of Queensland, Brisbane, QLD 4072 Australia; 4 AgResearch Grasslands Research Centre, PB 11008 Palmerston North, New Zealand; 5 School of Biomedical Sciences, The University of Queensland, Brisbane, QLD 4072 Australia

**Keywords:** Quantitative trait loci, Plant breeding, Plant genetics, Genetic variation

## Abstract

Plant breeders are supported by a range of tools that assist them to make decisions about the conduct or design of plant breeding programs. Simulations are a strategic tool that enables the breeder to integrate the multiple components of a breeding program into a number of proposed scenarios that are compared by a range of statistics measuring the efficiency of the proposed systems. A simulation study for the trait growth score compared two major strategies for breeding forage species, among half-sib family selection and among and within half-sib family selection. These scenarios highlighted new features of the QuLine program, now called QuLinePlus, incorporated to enable the software platform to be used to simulate breeding programs for cross-pollinated species. Each strategy was compared across three levels of half-sib family mean heritability (0.1, 0.5, and 0.9), across three sizes of the initial parental population (10, 50, and 100), and across three genetic effects models (fully additive model, a mixture of additive, partial and over dominance model, and a mixture of partial dominance and over dominance model). Among and within half-sib selection performed better than among half-sib selection for all scenarios. The new tools introduced into QuLinePlus should serve to accurately compare among methods and provide direction on how to achieve specific goals in the improvement of plant breeding programs for cross breeding species.

## Introduction

Cultivar development in a plant breeding program is a complex process involving a cyclical procedure over long periods. Choosing an appropriate breeding strategy is essential for a successful plant breeding program. However, it is difficult to evaluate the long-term effects of the chosen strategy, especially in the early phase of the program. Computer simulation can be a tool for plant breeders to efficiently examine breeding strategies for their breeding program and to make critical decisions accordingly (Sun et al. 2011). Computer simulation also allows, among other criteria, plant breeders to evaluate long-term effects of their breeding program. Information such as optimum parent population size, number of selection cycles needed to achieve maximum genetic potential, rates of accumulation of favorable alleles, rates of allele fixation, and the influence of breeding method on these outcomes, will help in developing breeding programs.

There are two types of computer simulations, deterministic and stochastic. Deterministic simulation for plant breeding programs is based on a set of mathematical equations developed from quantitative genetic theory. The output of this type of simulation is fully determined by the parameter values and initial conditions. Therefore, the output is bound by the assumptions of the equations used. Deterministic simulation has been implemented in DeltaGen (Jahufer and Luo 2018) to predict genetic gain and cost per selection cycle for a range of breeding strategies in forage species as a tactical tool using empirical data. However, due to the nature of the deterministic simulation, the output is only applied to the current cycle for the current initial conditions (e.g., current breeding population, current testing strategy, etc.).

On the other hand, stochastic simulation is designed to process variation and randomness of gene-to-phenotype relationship within the quantitative genetics framework. Stochastic simulation is more general than deterministic simulation because it is unconstrained by mathematical equations usually conditioned on some assumptions. Therefore, this type of simulation can be used to simulate whole breeding programs that are often too complex to be deterministically modelled. This type of simulation can be used as a strategic tool to compare multiple breeding strategies (Wang et al. 2003, 2009b) and to evaluate the impact of selection (Wang et al. 2007b, 2009a). It has been used to study the impact of genomic selection on the breeding program (Iwata and Jannink 2011; Lin et al. 2016). It has been used as a tactical tool to evaluate specific plant breeding questions, such as selection among parents, and to evaluate details of backcrossing strategies (Arief et al. 2014) and quantitative trait loci (QTL) introgression. For example, there are numerous challenges in selecting for multiple QTL within a breeding program, particularly if there are many QTL with small effects. When dealing with QTL introgression or pyramiding QTL, breeders need information that will allow them to decide how best to fit marker-assisted selection into a breeding pipeline. Computer simulations allow for the testing of multiple hypotheses in silico to determine the amount of resources necessary to convert elite lines with adapted backgrounds with multiple QTL and examine the maximum number of QTL that can be pyramided into a single genotype (Wang et al. 2007a).

QU-GENE is a software platform for stochastically simulating plant breeding programs (Podlich and Cooper 1998). QU-GENE uses *E*(*N*:*K*) models to simulate genotype-by-environment interactions (Podlich and Cooper 1998), where *E* is the number of environments, *N* is the number of genes, and *K* is the parameter designating the epistatic network. This model is a generalization of *NK* models (Kauffman 1993). This capability of QU-GENE to simulate genotype-by-environment interaction provides an advantage over other similar plant breeding simulation programs (e.g., Faux et al. 2016; Lin et al. 2016).

**Fig. 1:**
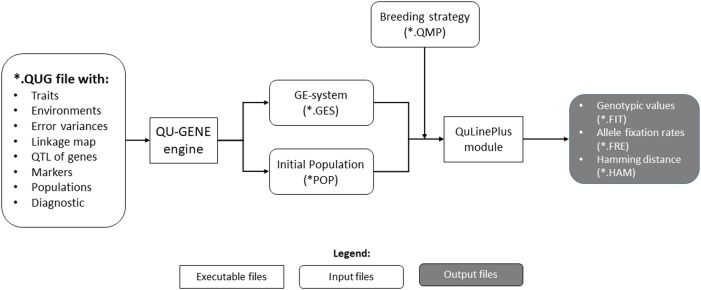
Workflow of QU-GENE

The QU-GENE platform consists of two components: An engine (referred to as the QU-GENE engine) to generate simulation input and breeding modules used to conduct simulations of breeding strategies. The QU-GENE engine is used to generate a genetic-by-environment system (GES). The breeding modules are used to simulate breeding strategies applied using this GES (Fig. 1). Currently there are three breeding modules available: QuLine (Wang and Dieters 2008a) for self-pollinating species; QuHybrid (Wang and Dieters 2008b) for hybrid development; QuMARS (Li and Wang 2011) for marker-assisted recurrent selection.

The objective of this paper is to introduce a new breeding module called QuLinePlus that has the additional capability to simulate open-pollinated species. This module is an extension of QuLine. We describe via examples how the new features in QuLinePlus are used to conduct simulation for breeding programs on open-pollinated populations with half-sib (HS) mating strategies. QU-GENE is free for academics and researchers in public institutions and available at http://sites.google.com/view/qu-gene.

## Materials and methods

### Theory behind QU-GENE

In quantitative genetics the phenotypic value of an individual is influenced by genes, environments and their interaction. The phenotypic values of individuals in a population measured for a trait in a number of environments can be represented by a standard factorial quantitative genetic model following Falconer and Mackay (1996) as

$$P = G + E + \left( {GE} \right) + R$$

In this model, *P* is the observed value of an individual, which is the sum of a genotype main effect *G*, an environment main effect *E*, an interaction effect (*GE*) between *G* and *E* plus an environment noise effect *R*. The genotype value *G* for an individual is the combined action of the alleles of all the genes affecting the trait in the population, and (*GE*) the combined action of the deviations from *G* of alleles in each of the environments.

Another form of the quantitative genetic model, the nested model

$$P = E + \left( {GE} \right) + R$$

emphasizes the differences in performance of genotypes in different environments. Again, (*GE*) is a composite of all the effects of the alleles of the genes affecting the trait. Variations and extensions of these models have been used in a mixed model framework (Cooper et al 2007) where all the terms except the grand mean are treated as random variables sampled from the breeding population. According to Cooper et al. (2007), the phenotypic value can also be considered as a combination of a ‘genetic signal’ component (*GE*), an ‘environment context’ component *E* and an ‘environmental noise’ component *R*. The genetic signal component and environment context can be modelled using *E*(*NK*) model outlined by Podlich and Cooper (1998), where *E* is the number of environments, *N* is the number of genes affecting a trait and *K* is the epistasis parameter which is the average number of other genes influenced by each gene. Hence, the phenotypic value for a genotype can be specified using the following form

$$P = E\left( {NK} \right) + R$$

where (*NK*) for each of *E* environments is the complete specification of all allelic effects of a set of *N* possibly interacting genes distributed across chromosomes using a recombination map. These *E*(*NK*) models are used to calculate the (*GE*) values for each genotype created by the QU-GENE program. The *NK* models for each environment can be fully specified or produced by sampling effects of alleles from appropriate distributions, or a combination of both.

This model is implemented in QU-GENE to simulate the genotype and genotype-by-environment interactions. In QU-GENE, the term environment refers to environmental types, such as mega-environments and R is the sum of among and within plot error (Wang and Dieters 2008a). Alternatively R can refer to the sum of the among and within family variances.

If the user wishes to calculate a numerator relationship matrix (NRM) or a genomic relationship matrix (GRM) to estimate simulated breeding values, it would be possible to do so by extracting the pedigrees of individual crosses generated throughout the simulation (NRM) or from the genotypes from a simulated or user-defined marker array (GRM).

### QuLinePlus

QuLinePlus is an extended version of QuLine and can be used to simulate open-pollinated species. The features of QuLinePlus are an update of QuLine such that the unit of manipulation (entity selected or crossed) becomes a family (population) rather than a fixed or pure line. Hence, breeding populations for fixed lines or clones become special cases of populations which are reduced to a set of complete (clones) or close to complete (pure lines) homogeneous genotypes. These new features are:

1. QuLinePlus can now specify crossing among multiple populations comprised of multiple individuals. Crosses are specified as mating amongst randomly selected individuals from within or among populations. Consequently, crossing block updates now replace all or a proportion of the original populations. Crosses among clones of fixed lines now become special cases when the family (population) consists of one or a few different genotypes.
2. QuLinePlus has an option to produce half-sib (HS) families. Half-sib families are created by bulking all genotypes derived from each female used in the crossing block. Self-pollination of female lines is excluded from the half-sib families. QuLinePlus still includes the three options of QuLine to produce: (a) a single family (population) by bulking all genotypes in the breeding program, (b) many families by bulking all genotypes derived from single plant or single family, (c) many families by separating all selected genotypes into different families.
3. The *.FIT file generated by QuLine reports the genotypic mean of the populations derived from each cycle of simulation. Since QuLine was programmed under the assumption that all lines derived from a breeding cycle would be a ‘fixed line’, each was represented by the value of one randomly selected individual. QuLinePlus now retains and uses all individuals in the final populations from a cycle to calculate the mean of the family for the *.FIT file.
4. Another option, ‘*polycross’* was added to the propagation type options. This option enables among family polycrosses to generate new half-sib family or synthetic population in any generation after the first initial crosses.
5. QuLinePlus has an extra option for updating the crossing block. This update has the ability to select among initial parents (populations) based on progeny testing.

In addition to the new features, an R Shiny graphical user interface is created to help users run the QU-GENE simulation (Fig. 1). A version of this interface is available from http://sites.google.com/view/qu-gene/download.

### Case studies in forage breeding

QuLinePlus was used to simulate two breeding strategies commonly used for cross-pollinating forage species after Casler and Brummer (2008). The two strategies are among half-sib family selection (AHS) for among HS family selection, and among and within half-sib family selection (AWHS) for among and within HS family selection. These scenarios demonstrate the new facility of QuLinePlus to create HS families.

#### Breeding strategies

**Fig. 2:**
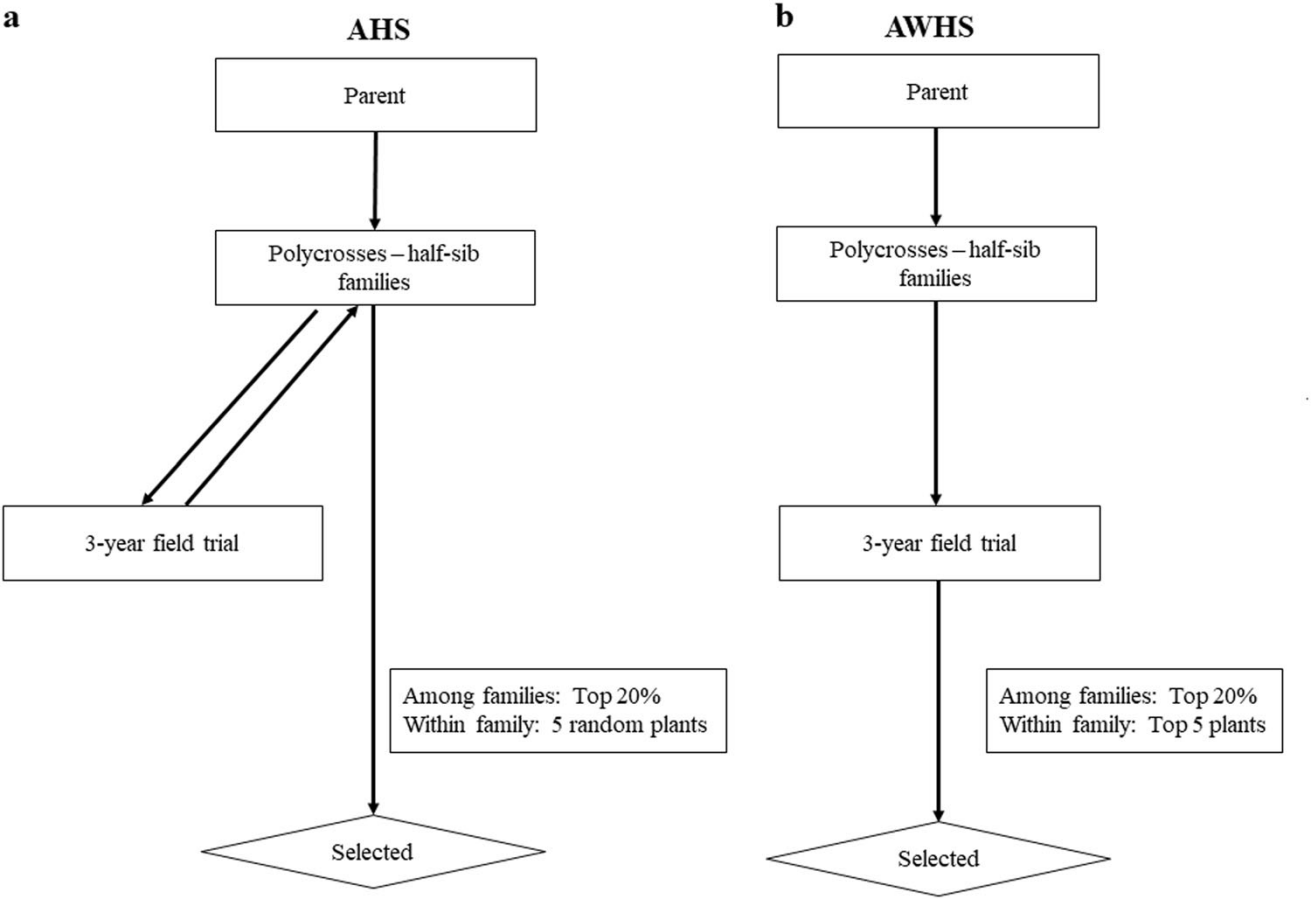
Two breeding strategies for forage species: **a** among half-sib family selection (AHS) and **b** among and within half-sib family selection (AWHS)

HS families derived from the female parents from all the pairwise crosses among a set of families. These HS families were then tested for three years in three locations using three replications with a plot size of 30 plants. Based on these trials, 20% of HS families with the best growth score were advanced to the next cycle. To restore the initial number of parents, either a random five in AHS or best five in AWHS were selected from each HS family (Fig. 2). These selected plants were then used as parents for HS family of the next cycle. Both strategies were run for 50 cycles.

#### Genotype-by-environment system

These two breeding strategies were compared under 27 genotype-by-environment systems: three genetic effects models, three levels of HS family mean heritability, and three sizes of parental populations. In total, there were 54 simulation scenarios with each run 100 times (Supplementary Table 1).

A total of 63 previously reported QTL for growth scores (Faville et al. 2012) were distributed on the linkage map constructed from Khaembah et al. (2013) and Sartie et al. (2011). This linkage map also included 194 markers. Growth score is a major trait collected in forage breeding programs. Three genetic effects models were simulated for these QTL: (1) fully additive with no dominance (referred to as the additive model), (2) a mixture of additive, partial and over dominance with 50% additive (referred to as the additive-dominance model), and (3) a mixture of partial and over dominance (referred to as the dominance model). No epistasis networks were considered. All QTL effects were randomly drawn from a normal distribution with the mean of zero and standard deviation of one (Wang and Dieters 2008a).

Three levels of HS family mean heritability (0.1, 0.5, and 0.9) were used to generate phenotypic data. These heritabilities reflected the range of HS family mean heritability from three-year field trials.

Three sizes of the parental populations (10, 50, and 100) were randomly generated using QU-GENE engine with allele frequency for all loci set to be 0.5. These initial populations were used to create the initial HS families. Parental populations were set to have equal fractions of homozygous and heterozygous loci, randomly assigned with each simulation run.

#### Simulation outputs

Three criteria are commonly used to compare the two breeding strategies from QU-GENE simulation: genetic gain, Hamming distance, and allele fixation rates. Genetic gain is calculated as the difference in genotypic mean (*.FIT) between cycles. This genetic gain can be expressed as a percentage to remove scaling factors to enable comparisons among simulation scenarios (Wang and Dieters 2008a). Cumulative genetic gain can also be calculated across cycles. In this study, two criteria were used to compare simulation results: 90% cumulative gain (ΔG90) and the number of cycles required to achieve ΔG90.

Hamming distance (*.HAM) is a compound measure of the number of unfavorable alleles that are necessary to be substituted by favorable alleles to reach an ideal genotype (He et al. 2004). A smaller value for Hamming distance means that the selected population is closer to the ideal genotypes. Allele fixation rates (*.FRE) are calculated favorable and non-favorable alleles for each cycle (Wang and Dieters 2008a). Hamming distance and allele fixation rates are reflecting the impact of selection on genetic diversity.

## Results

### Additive model

**Fig. 3:**
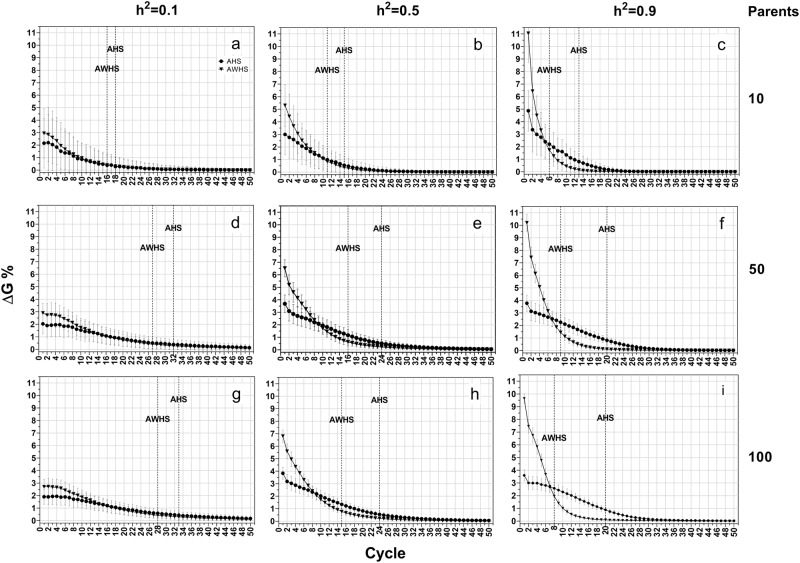
Average genetic gain (ΔG%) of growth score for among half-sib family selection (AHS) and among and within half-sib family selection (AWHS) under an additive genetic model for three levels of heritability (0.1, 0.5, and 0.9, row-wise) and three initial parent population sizes (10, 50 and 100, column-wise). Dotted vertical lines represent the breeding cycle at which 90% of the cumulative genetic gain (ΔG90) is achieved

Under the additive model, AWHS always had more genetic gain than AHS, especially in early cycles across different levels of heritability and different sizes of parental populations (Fig. 3). In both breeding strategies, a higher level of heritability resulted in faster achievement of ΔG90 (Fig. 3, dotted line). However, a higher level of heritability increased the gap between AHS and AWHS. For example, there was only one cycle difference between AHS and AWHS to achieve ΔG90 for heritability 0.1 (Fig. 3a), but seven cycles difference for heritability 0.9 (Fig. 3d). The changes in heritability had a larger impact on genetic gain of AWHS than AHS, especially for the first cycle. For example, the genetic gain for the first cycle in AWHS increased by 2.5% when heritability increased from 0.1 to 0.5, whereas genetic gain for AHS only increased by 1% (Fig. 3a, b). This genetic gain seemed less affected by the change in population size (Fig. 3, column-wise). While increasing population size from 10 to 50 improved genetic gain, there was no further improvement by increasing population size from 50 to 100.

**Fig. 4:**
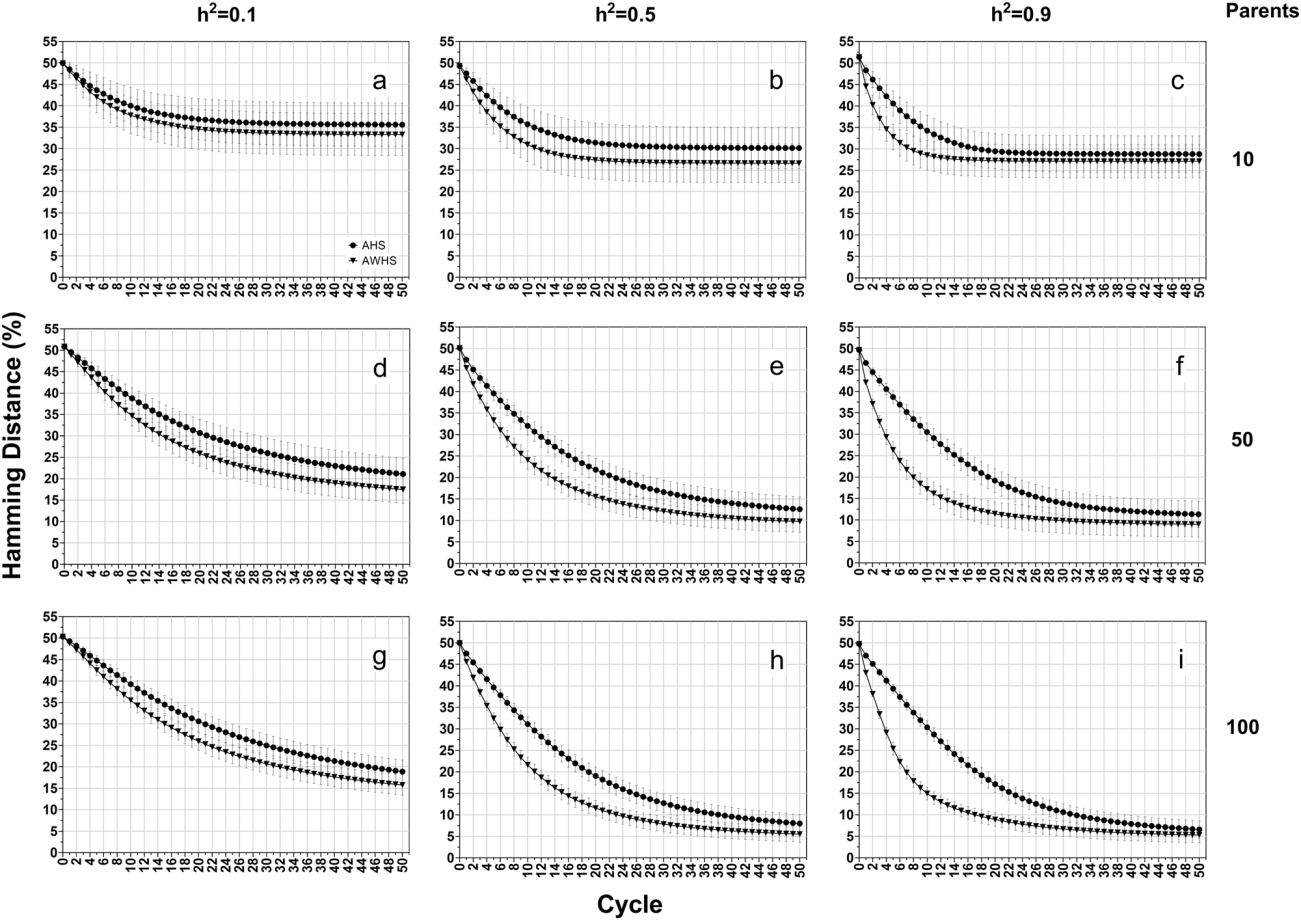
Hamming distance of forage growth score for among half-sib family selection (AHS) and among and within half-sib family selection (AWHS) under an additive genetic model for three levels of heritability (0.1, 0.5, and 0.9, row-wise) and three initial parent population sizes (10, 50 and 100, column-wise)

In contrast to genetic gain, Hamming distance (Fig. 4) and allele fixation rates (Supplementary Figure 1) were more affected by population sizes than by heritability levels. Increasing population size resulted in a lower value of Hamming distance (Fig. 4, column-wise) and allele fixation rate for both favorable and non-favorable alleles (Supplementary Figure 1). As with genetic gain, the gap between AHS and AWHS increased with the increasing level of heritability (Fig. 4 and Supplementary Figure 1, row-wise).

### Additive-dominance model

**Fig. 5:**
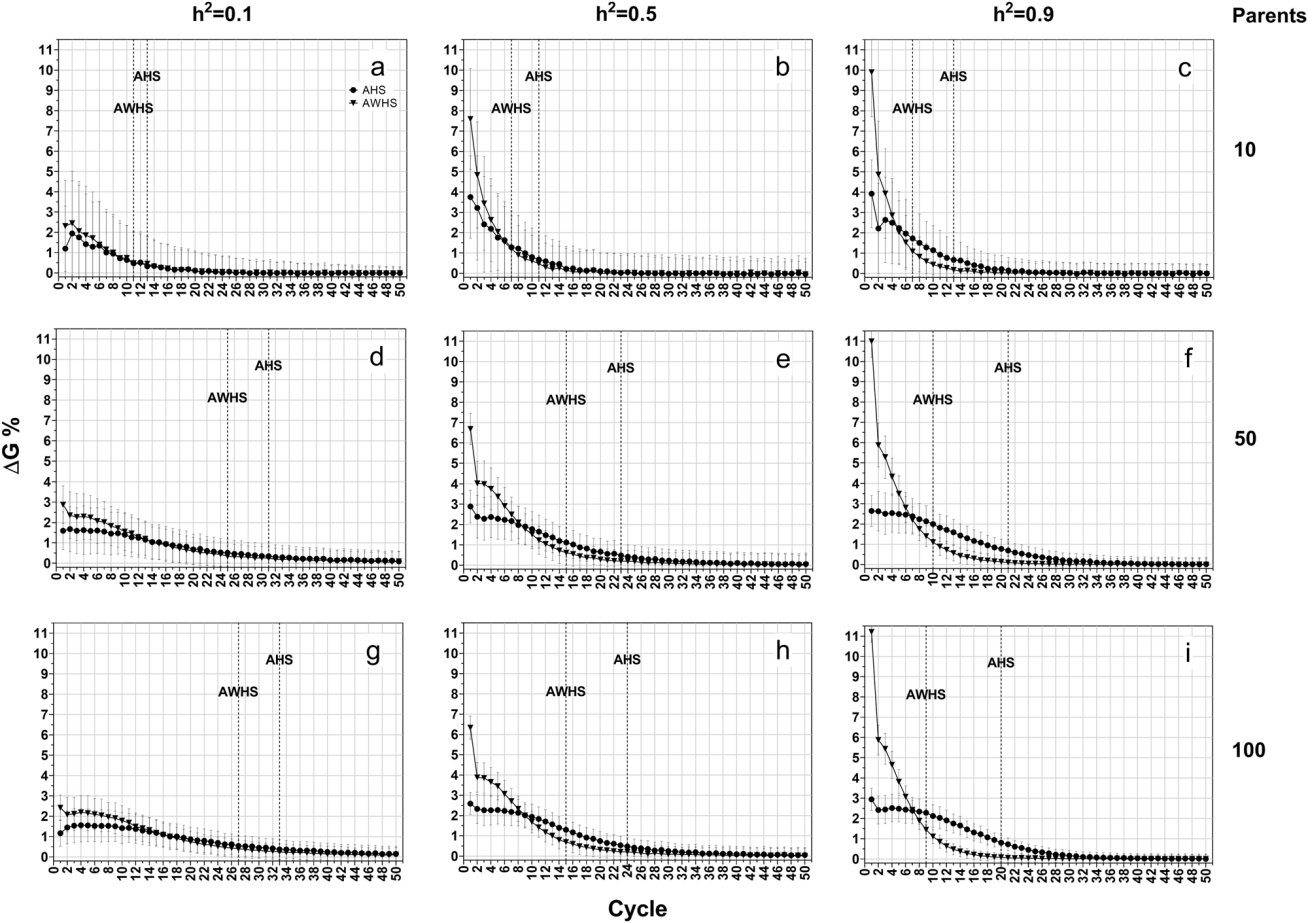
Average genetic gain (ΔG%) of growth score for among half-sib family selection (AHS) and among and within half-sib family selection (AWHS) under an additive-dominance genetic model (a mixture of additive, partial and over dominance genetic models) for three levels of heritability (0.1, 0.5, and 0.9, row-wise) and three initial parent population sizes (10, 50 and 100, column-wise). Dotted vertical lines represent the breeding cycle at which 90% of the cumulative genetic gain (ΔG90) is achieved

**Fig. 6:**
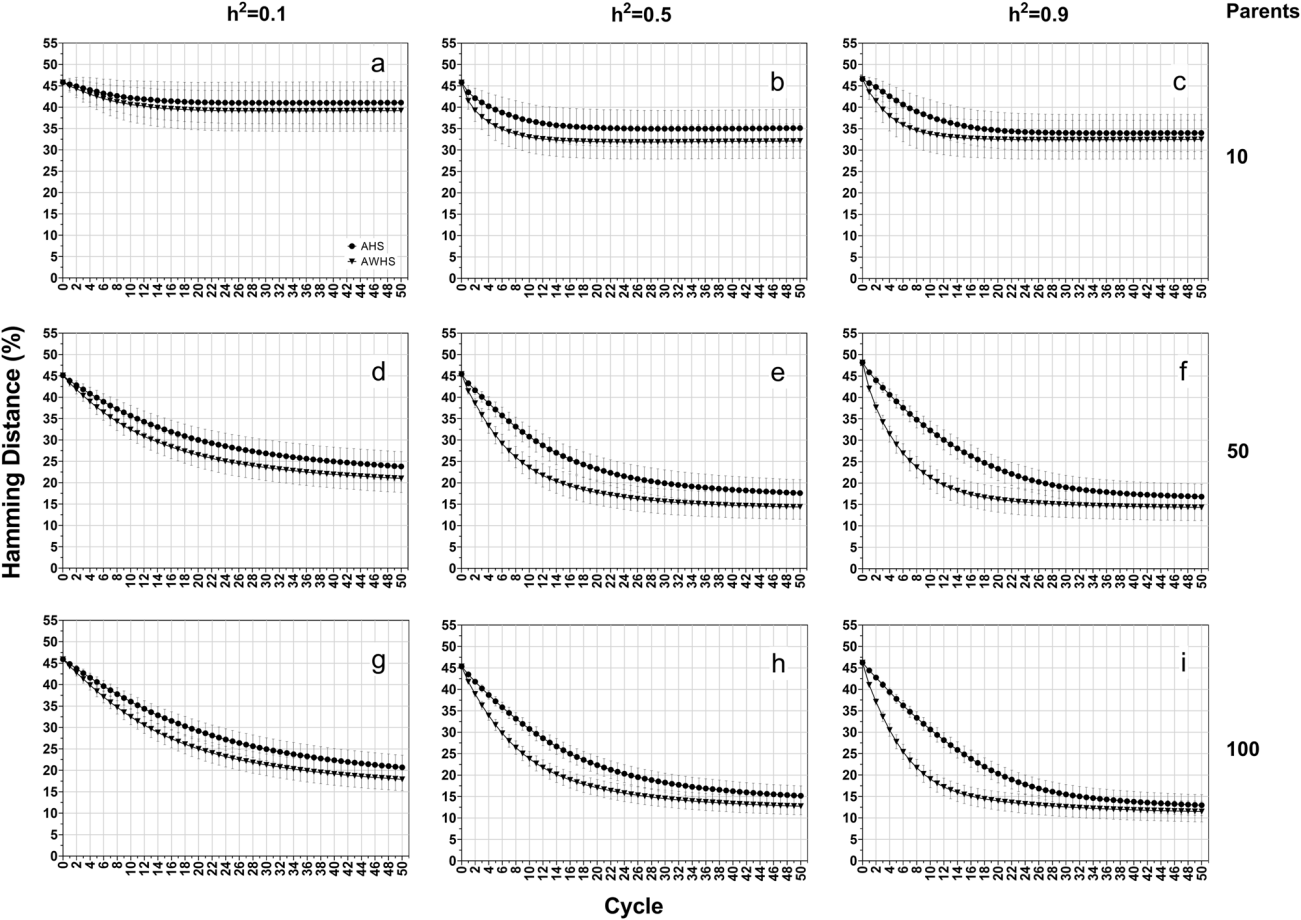
Hamming distance of forage growth score for among half-sib family selection (AHS) and among and within half-sib family selection (AWHS) under an additive-dominance genetic model (a mixture of additive, partial and over dominance genetic models) for three levels of heritability (0.1, 0.5, and 0.9, row-wise) and three initial parent population sizes (10, 50 and 100, column-wise)

Under the additive-dominance model, AWHS still had more genetic gain than AHS (Fig. 5). However, the differences were smaller than those observed under the additive model (Fig. 3). This genetic model also resulted in a larger value of Hamming distance (Fig. 6) compared to that from the additive model (Fig. 4). However, there was almost no difference in the pattern of allele fixation rates between the additive-dominance model (Supplementary Figure 2) and the fully additive model (Supplementary Figure 1), except that the difference in allele fixation rates between favorable and non-favorable alleles was smaller than previously observed under the additive genetic model.

### Dominance model

**Fig. 7:**
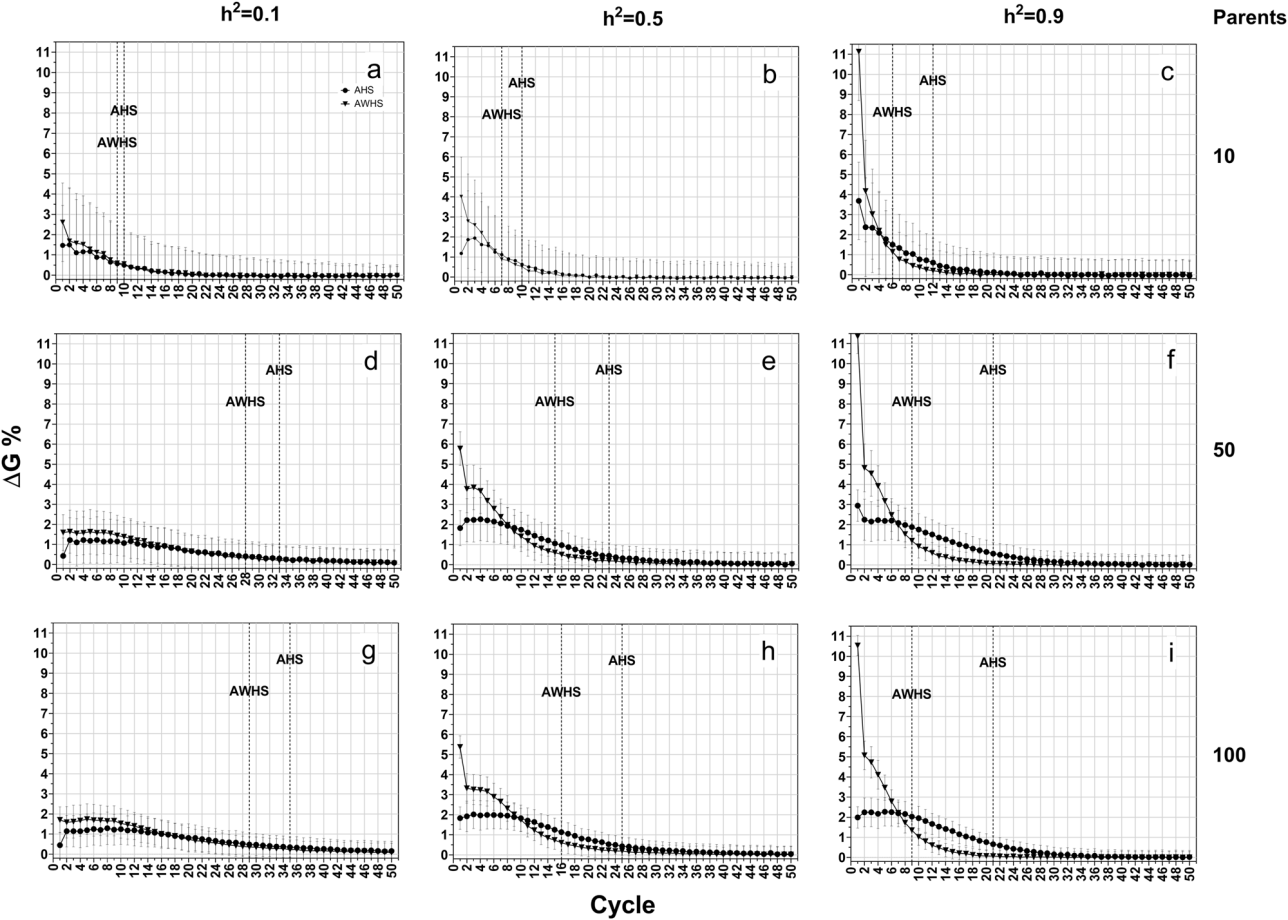
Average genetic gain (ΔG%) of growth score for among half-sib family selection (AHS) and among and within half-sib family selection (AWHS) under a dominance genetic model (a mixture of partial and over dominance genetic models) for three levels of heritability (0.1, 0.5, and 0.9, row-wise) and three initial parent population sizes (10, 50 and 100, column-wise). Dotted vertical lines represent the breeding cycle at which 90% of the cumulative genetic gain (ΔG90) is achieved

**Fig. 8:**
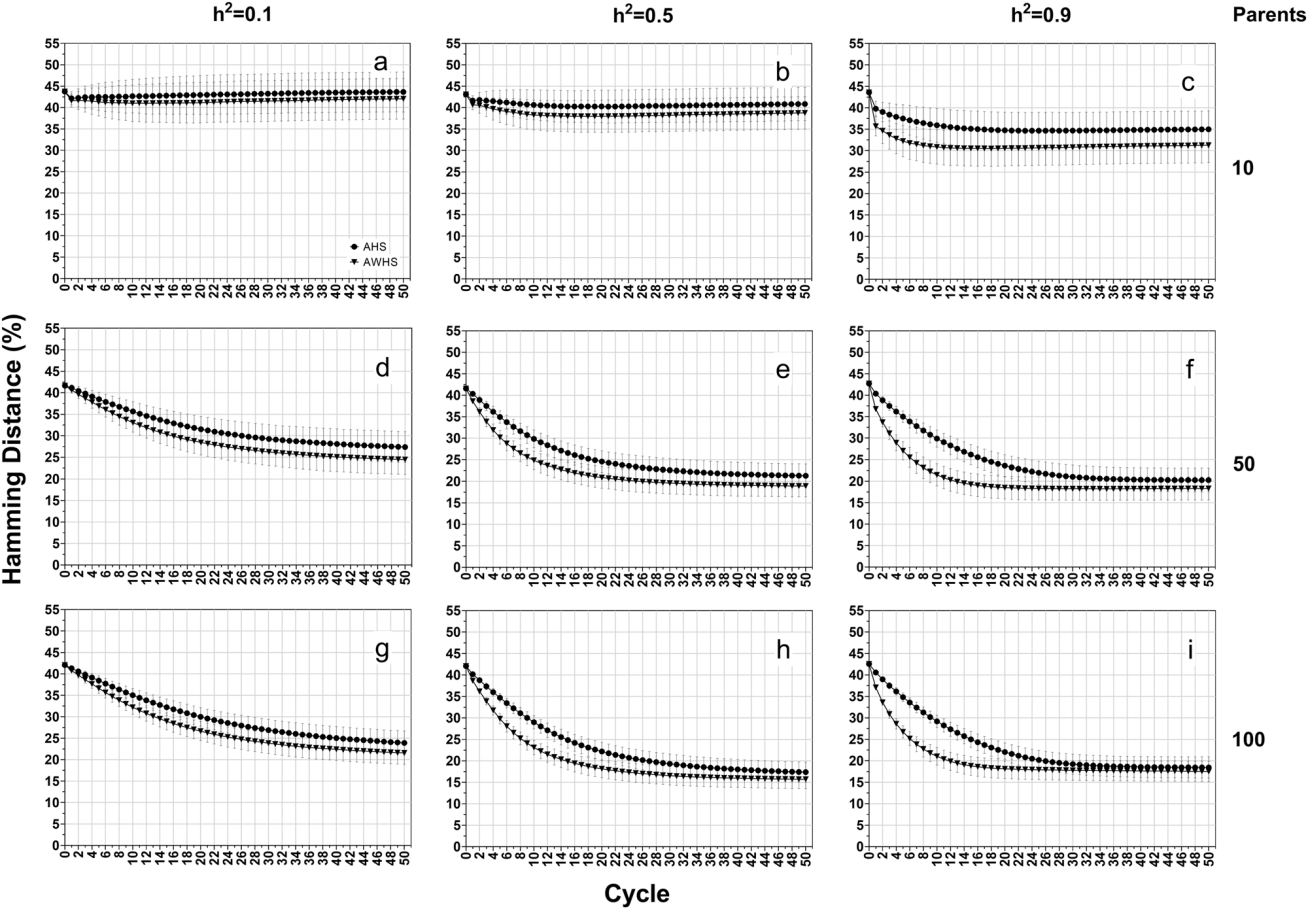
Hamming distance of forage growth score for among half-sib family selection (AHS) and among and within half-sib family selection (AWHS) under a dominance genetic model (a mixture of partial and over dominance genetic models) for three levels of heritability (0.1, 0.5, and 0.9, row-wise) and three initial parent population sizes (10, 50 and 100, column-wise)

Genetic gain under the dominance genetic model (Fig. 7) showed similar patterns as those observed under the additive-dominance model (Fig. 5). The Hamming distances from this model (Fig. 8) were larger than those from the two previous genetic effects models. These results indicated that the selected populations under this genetic model were further away from ideal genotypes. While the patterns of allele fixation rates were similar to those observed in the other two genetic effects models (Supplementary Figure 3), the difference in fixation rate between favorable and non-favorable were becoming smaller. The allele fixation rates for the favorable and non-favorable alleles were almost the same under heritability of 0.1 and population size of 10 (Supplementary Figure 3a).

### Comparisons

**Fig. 9:**
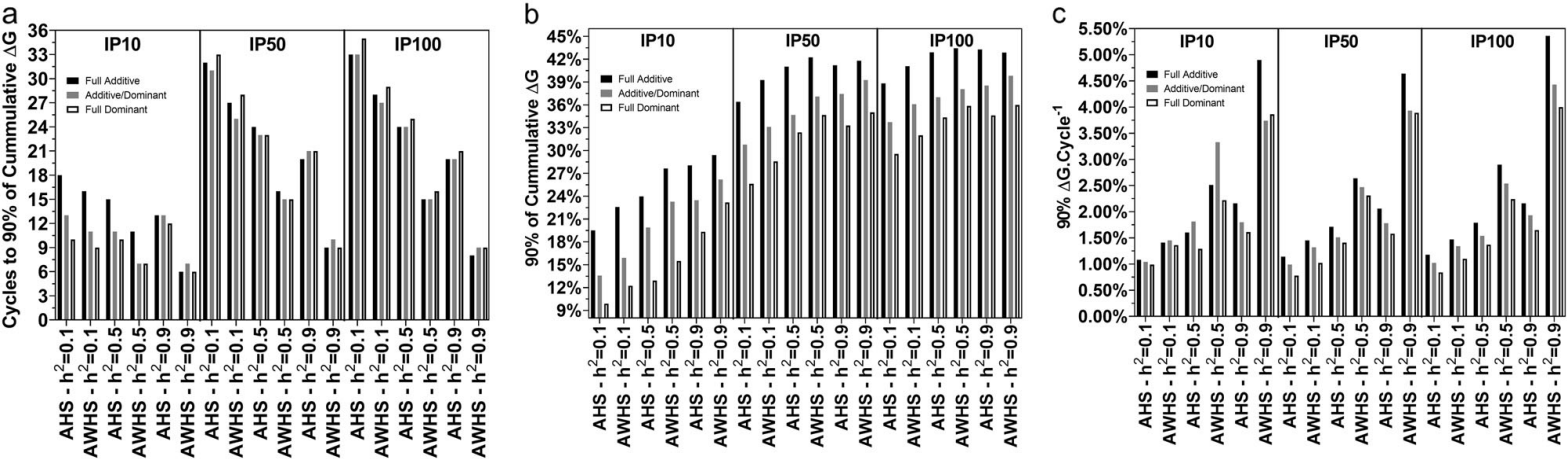
Summary statistics of the simulations performed using QuLinePlus across the three genetic models (additive, additive-dominance and dominance), three initial parental population sizes (10, 50, and 100), and three levels of heritability (0.1, 0.5, and 0.9) for among half-sib family selection (AHS) and among and within half-sib family selection (AWHS). **a** The number of cycles to achieve 90% of the cumulative genetic gain (ΔG90). **b** The 90% cumulative genetic gain (ΔG90). **c** The 90% cumulative gain per cycle. These values are mean values across 100 simulation runs

On average, AWHS always performed better than AHS (Fig. 9). Under AWHS, ΔG90 was achieved faster than that under AHS (Fig. 9a). The values of ΔG90 from AWHS were also larger than those from AHS (Fig. 9b). Therefore, the result was larger ΔG90 per cycle for AWHS (Fig. 9c). For both strategies, increasing population size from 10 to 50 improved ΔG90, but there was no advantage by increasing from 50 to 100 (Fig. 9b). However, increasing population size from 10 to 50 also resulted in increasing the number of cycles to achieve ΔG90 (Fig. 9a).

Within each breeding strategy, the additive model resulted in larger ΔG90 (Fig. 9b), but required a longer time to achieve it (Fig. 9a). On the other hand, the dominance model resulted in lower ΔG90 in fewer cycles. The pattern of ΔG90 across the three genetic effects models was similar across three levels of heritability and the three population sizes (Fig. 9b). However, the pattern of the number of cycles to achieve ΔG90 across the three genetic effects models changed as the heritability and population size increased (Fig. 9a). The differences in the number of cycles among the genetic effects models became smaller as heritability increased in the small population (10) or as population increased from 50 to 100.

At the end of 50 cycles, the additive model reached a minimum Hamming distance of 5% (Fig. 4i), the additive-dominance model reached a minimum of 10% (Fig. 6i), and the dominance model reached a minimum of 18% (Fig. 8i). Under the three genetic effects models, the fixation rates for both favorable and non-favorable alleles were always higher in AWHS than AHS (Supplementary Figures 1, 2 and 3). For both strategies, the dominance model resulted in the lowest and the highest fixation rate for favorable and non-favorable alleles, respectively. Patterns of inbreeding can be deduced from changes in non-favorable allele fixation. At a small population size, complete fixation of non-favorable alleles occurs at a faster rate. As expected, larger population size lead to lower and slower accumulation of non-favorable alleles.

## Discussion

By using a HS population module in QuLinePlus, we have revealed a range of patterns for forage breeding programs and for other HS population-improved species. Overall, AWHS resulted in higher rates of genetic gain than AHS. Depending on the heritability level, AWHS provided a greater advantage than AHS. Under HS breeding, 75% of the genetic variance is contained within families and the remaining 25% is found among families (Hallauer et al. 2010). AWHS utilizes both between and within family variances, while AHS only utilizes between family selections. Therefore, AWHS is expected to perform better than AHS.

AWHS is expected to perform better than AHS for low levels of heritability as better individuals are selected within families rather than at random. Lower heritability means larger environmental noise in phenotypic values. Therefore, for phenotypic selection, some inferior individuals can be selected in AHS due to chance.

As expected, the relationship changes as heritability becomes larger, whereby environmental noise is minimized and both AHS and AWHS receive an advantage under any of the genetic effects models of additive, additive-dominance or dominance. It is important to note that the difference in ΔG90 becomes smaller between the two strategies as heritability increases, particularly under full additivity (Fig. 9b). In contrast, the difference in ΔG90 per cycle due to changes in heritability between AHS and AWHS is more evident at larger population sizes, particularly under the dominance model (Fig. 9c), and this is due to a larger number of cycles required to reach ΔG90 using AHS. Based on expected gain per cycle calculated for breeder’s equation (Falconer and Mackay 1996), Casler and Brummer (2008) concluded that AWHS resulted in far superior performance than AHS when heritability was moderate (0.5) to high (1) but only did half as well as AHS when heritability was below 0.2 under 20% selection intensity.

The ΔG90 per cycle (Fig. 9c) is a cost-benefit criterion that is useful for making comparisons. For example, under full additive model for 100 initial populations and heritability of 0.5, ΔG90 for AWHS and AHS were 43 and 42%, respectively (Fig. 9b). Based on this value, it seems that there was no significant difference between AWHS and AHS. However, it required 14 cycles of AWHS and 23 cycles of AHS to achieve their respective ΔG90. Therefore, based on ΔG90 per cycle, AWHS was more effective than AHS. Within AWHS, increasing the population size from 10 to 50 seems to provide an increase of roughly 15–20% in ΔG90 (Fig. 9b). However, it also leads to an increase of around 15–20 cycles to achieve this ΔG90 (Fig. 9a). Therefore, based on ΔG90 per cycle there was not much advantage to increasing the initial population size from 10 to 50 (Fig. 9c). There were also costs associated with creating and testing additional HS families that were not taken into consideration. If these additional costs are considered, increasing the size of the initial population from 10 to 50 may provide no advantage.

Computer simulations have been used in crops such as wheat and rice to make strategic decisions on steering actual breeding programs and populations (Wang 2011). Lin et al. (2016) simulated genetic gain in ryegrass and compare a model phenotypic selection strategy with a commercial breeding program modified for genomic selection and Faville et al. (2018) have demonstrated it empirically for genomic selection. Using computer simulations could provide insight on the impact of selections under different genetic effects models.

In this study, AWHS was shown to perform better than AHS across the three genetic effects models. However, the full dominance model always resulted in lower ΔG90 than the full additive model (Fig. 9b), but required fewer or similar number of cycles to achieve that ΔG90 (Fig. 9a). Lower ΔG90 under the dominance model could be due to a higher fixation rate of non-favorable alleles and a lower fixation rate of favorable alleles (Supplementary Figure 3).

## Conclusions

The purpose of this article is to introduce QuLinePlus for simulating breeding programs for open-pollinated species. We compared two commonly used HS breeding strategies using theoretical models. The simulation results using QuLinePlus confirmed the superiority of AWHS over AHS across different levels of heritability, genetic effects models, and population sizes.

The simulations presented here test strategies that operate under a closed system between cycles, that is, no new genetic variation is introduced among the initial parents at the beginning of a new cycle. For example, the effects of reciprocal recurrent selection schemes, where variation generated at the end of a cycle is introduced into another population and vice-versa can't be performed in simultaneous runs. In future, it would be useful to confirm the superiority of AWHS using other models that also include genomic selection.

### Data archiving

Data available from the Dryad Digital Repository: 10.5061/dryad.7368cc2.

## Electronic supplementary material

Supplementary Table 1

Supplementary Figure 1

Supplementary Figure 2

Supplementary Figure 3

## References

[CR1] Arief VN, DeLacy IH, Dieters MJ, Basford KE (2014). Application of marker-trait association profiles in simulating plant breeding strategies 15th Australasian Plant Breeding Conference.

[CR2] Casler MD, Brummer EC (2008). Theoretical expected genetic gain for among-and-within-family selection methods in perennial forage crops. Crop Sci.

[CR3] Cooper M, Podlich DW, Luo L, Varshney RK, Tuberosa R (2007). Modeling QTL effects and MAS in plant breeding. Genomics-assisted crop improvement: Vol. 1: Genomics approaches and platforms.

[CR4] Falconer DS, Mackay TF (1996). Introduction to quantitative genetics.

[CR5] Faux AM, Gorjanc G, Gaynor RC, Battagin M, Edwards SM, Wilson DL, Hearne SJ, Gonen S, Hickey JM (2016). AlphaSim: Software for Breeding Program Simulation. Plant Genome 9: 10.3835/plantgenome2016.3802.0013. 10.3835/plantgenome2016.02.0013 27902803

[CR6] Faville MJ, Ganesh S, Cao M, Jahufer MZZ, Bilton TP, Easton HS, Ryan DL (2018). Predictive ability of genomic selection models in a multi-population perennial ryegrass training set using genotyping-by-sequencing. Theor Appl Genet.

[CR7] Faville MJ, Jahufer MZZ, Hume DE, Cooper BM, Pennell CGL, Ryan DL, Easton HS (2012). A quantitative trait locus analysis of herbage biomass production in perennial ryegrass. NZ J Agric Res.

[CR8] Hallauer AR, Carena MJ, Miranda JB (2010). Means and Variances. Quantitative genetics in maize breeding.. Iowa State University Pres. Ames..

[CR26] He MX, Petukhov SV, Ricci PE (2004). Genetic code, hamming distance and stochastic matrices. Bulletin of Mathematical Biology.

[CR9] Iwata H, Jannink JL (2011). Accuracy of Genomic Selection Prediction in Barley Breeding Programs: A Simulation Study Based On the Real Single Nucleotide Polymorphism Data of Barley Breeding Lines. Crop Sci.

[CR10] Jahufer M. Z. Z., Luo Dongwen (2018). DeltaGen: A Comprehensive Decision Support Tool for Plant Breeders. Crop Science.

[CR11] Kauffman SA (1993). The origins of order: self-organization and selection in evolution.

[CR12] Khaembah EN, Irving LJ, Thom ER, Faville MJ, Easton HS, Matthew C (2013). Leaf Rubisco turnover in a perennial ryegrass (*Lolium perenne* L.) mapping population: genetic variation, identification of associated QTL, and correlation with plant morphology and yield. J Exp Bot.

[CR13] Li H, Wang J (2011). Simulation modeling in crop breeding. J Indian Soc Agric Stat.

[CR14] Lin Zibei, Cogan Noel O. I., Pembleton Luke W., Spangenberg German C., Forster John W., Hayes Ben J., Daetwyler Hans D. (2016). Genetic Gain and Inbreeding from Genomic Selection in a Simulated Commercial Breeding Program for Perennial Ryegrass. The Plant Genome.

[CR15] Podlich DW, Cooper M (1998). QU-GENE: a simulation platform for quantitative analysis of genetic models. Bioinformatics.

[CR16] Sartie AM, Matthew C, Easton HS, Faville MJ (2011). Phenotypic and QTL analyses of herbage production-related traits in perennial ryegrass (*Lolium perenne* L.). Euphytica.

[CR17] Sun X, Peng T, Mumm RH (2011). The role and basics of computer simulation in support of critical decisions in plant breeding. Mol Breed.

[CR18] Wang, J (2011). QuMARS, A QU-GENE application module that simulates marker assisted recurrent selection. Version 1.0. User’s Manual.

[CR19] Wang J, Chapman SC, Bonnett DG, Rebetzke GJ (2009). Simultaneous selection of major and minor genes: use of QTL to increase selection efficiency of coleoptile length of wheat (*Triticum aestivum* L.). Theor Appl Genet.

[CR20] Wang J, Chapman SC, Bonnett DG, Rebetzke GJ, Crouch J (2007). Application of population genetic theory and simulation models to efficiently pyramid multiple genes via marker-assisted selection. Crop Sci.

[CR21] Wang J, Dieters D (2008a). QuLine, A software that simulates breeding programs for developing inbred lines. Version 2.1. User’s Manual.

[CR22] Wang J, Dieters M (2008b). QuHybrid, A QU-GENE application module that simulates breeding programs for developing hybrids. Version 1.0. User’s Manual.

[CR23] Wang J, van Ginkel M, Podlich D, Ye G, Trethowan R, Pfeiffer W, DeLacy IH, Cooper M, Rajaram S (2003). Comparison of two breeding strategies by computer simulation. Crop Sci.

[CR24] Wang J, Singh RP, Braun HJ, Pfeiffer WH (2009). Investigating the efficiency of the single backcrossing breeding strategy through computer simulation. Theor Appl Genet.

[CR25] Wang J, Wan X, Li H, Pfeiffer WH, Crouch J, Wan J (2007). Application of identified QTL-marker associations in rice quality improvement through a design-breeding approach. Theor Appl Genet.

