# Supplementary material for: QuLinePlus: extending plant breeding strategy and genetic model simulation to cross-pollinated populations—case studies in forage breeding
Source: Heredity (Edinb). 2018 Oct 27;122(5):684–95. doi: 10.1038/s41437-018-0156-0 (PMC6461948; doi:10.1038/s41437-018-0156-0)
Supplement: Supplementary file 1 — Supplementary Table 1 [file 41437_2018_156_MOESM1_ESM.docx]

**Supplementary Table 1. Structure of the simulations performed using the new features of QuLinePlus**

| **No.** | **Gene** | **Initial** | **Heritability** | **Strategies** | **No.** | **Gene** | **Initial** | **Heritability** | **Strategies** | **No.** | **Gene** | **Initial** | **Heritability** | **Strategies** |
| --- | --- | --- | --- | --- | --- | --- | --- | --- | --- | --- | --- | --- | --- | --- |
|  | **Model** | **Parents** |  |  |  | **Model** | **Parents** |  |  |  | **Model** | **Parents** |  |  |
| 1 | Additive | 10 | 0.1 | AHS | 19 | Additive-Dominant | 10 | 0.1 | AWHS | 37 | Dominant | 10 | 0.1 | AWHS |
| 2 |  |  | 0.5 |  | 20 |  |  | 0.5 |  | 38 |  |  | 0.5 |  |
| 3 |  |  | 0.9 |  | 21 |  |  | 0.9 |  | 39 |  |  | 0.9 |  |
| 4 |  | 50 | 0.1 |  | 22 |  | 50 | 0.1 |  | 40 |  | 50 | 0.1 |  |
| 5 |  |  | 0.5 |  | 23 |  |  | 0.5 |  | 41 |  |  | 0.5 |  |
| 6 |  |  | 0.9 |  | 24 |  |  | 0.9 |  | 42 |  |  | 0.9 |  |
| 7 |  | 100 | 0.1 |  | 25 |  | 100 | 0.1 |  | 43 |  | 100 | 0.1 |  |
| 8 |  |  | 0.5 |  | 26 |  |  | 0.5 |  | 44 |  |  | 0.5 |  |
| 9 |  |  | 0.9 |  | 27 |  |  | 0.9 |  | 45 |  |  | 0.9 |  |
| 10 |  | 10 | 0.1 | AWHS | 28 |  | 10 | 0.1 | AHS | 46 |  | 10 | 0.1 | AHS |
| 11 |  |  | 0.5 |  | 29 |  |  | 0.5 |  | 47 |  |  | 0.5 |  |
| 12 |  |  | 0.9 |  | 30 |  |  | 0.9 |  | 48 |  |  | 0.9 |  |
| 13 |  | 50 | 0.1 |  | 31 |  | 50 | 0.1 |  | 49 |  | 50 | 0.1 |  |
| 14 |  |  | 0.5 |  | 32 |  |  | 0.5 |  | 50 |  |  | 0.5 |  |
| 15 |  |  | 0.9 |  | 33 |  |  | 0.9 |  | 51 |  |  | 0.9 |  |
| 16 |  | 100 | 0.1 |  | 34 |  | 100 | 0.1 |  | 52 |  | 100 | 0.1 |  |
| 17 |  |  | 0.5 |  | 35 |  |  | 0.5 |  | 53 |  |  | 0.5 |  |
| 18 |  |  | 0.9 |  | 36 |  |  | 0.9 |  | 54 |  |  | 0.9 |  |

Each simulation scenario was conducted for 10 random models and 100 runs; AHS = among half-sib family selection; AWHS = among and within half-sib family selection
