# Supplementary figures and images for: QuLinePlus: extending plant breeding strategy and genetic model simulation to cross-pollinated populations—case studies in forage breeding
Source: Heredity (Edinb). 2018 Oct 27;122(5):684–95. doi: 10.1038/s41437-018-0156-0 (PMC6461948; doi:10.1038/s41437-018-0156-0)

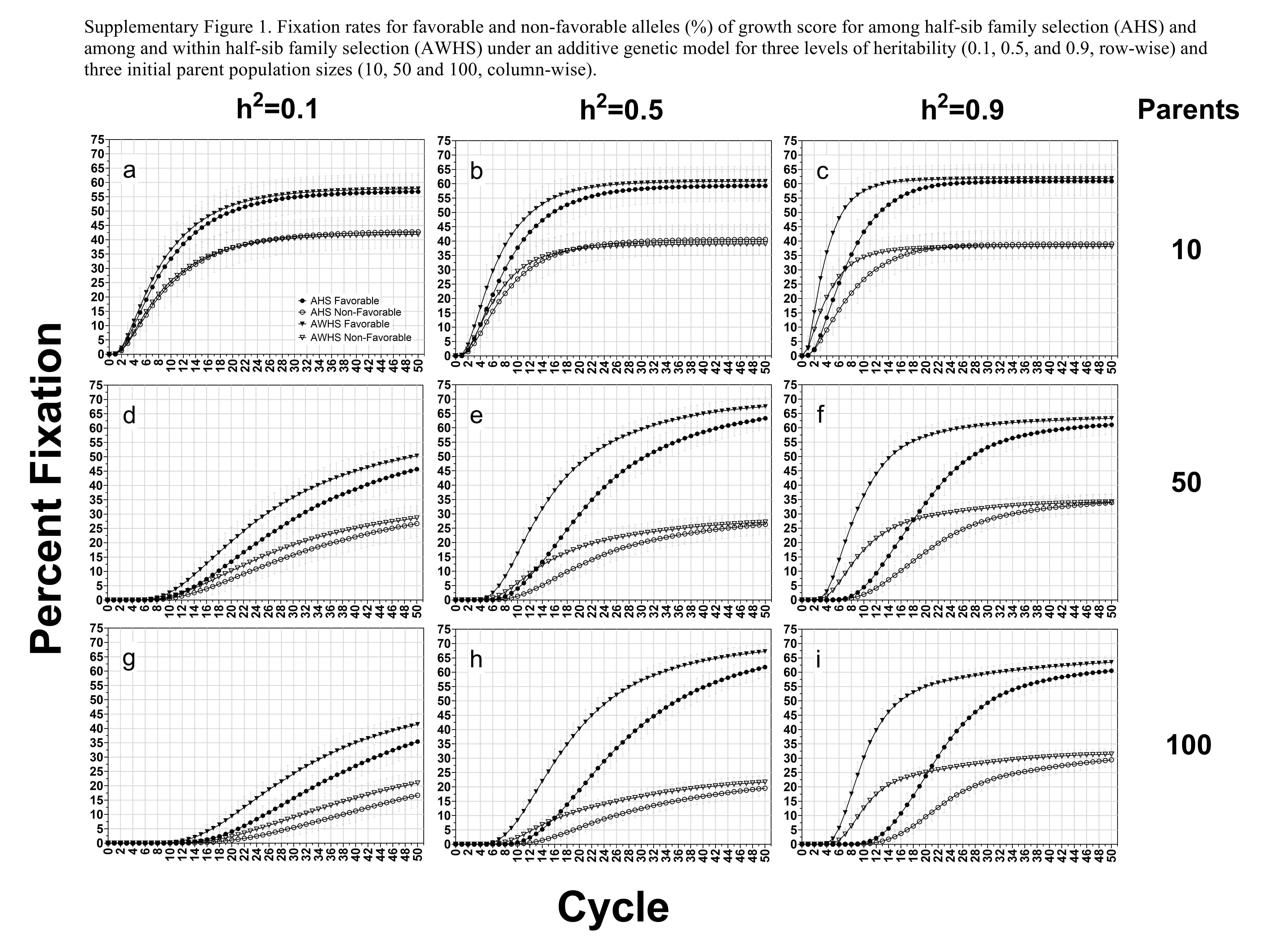

Supplement: Supplementary file 2 — Supplementary Figure 1 [file 41437_2018_156_MOESM2_ESM.tif]

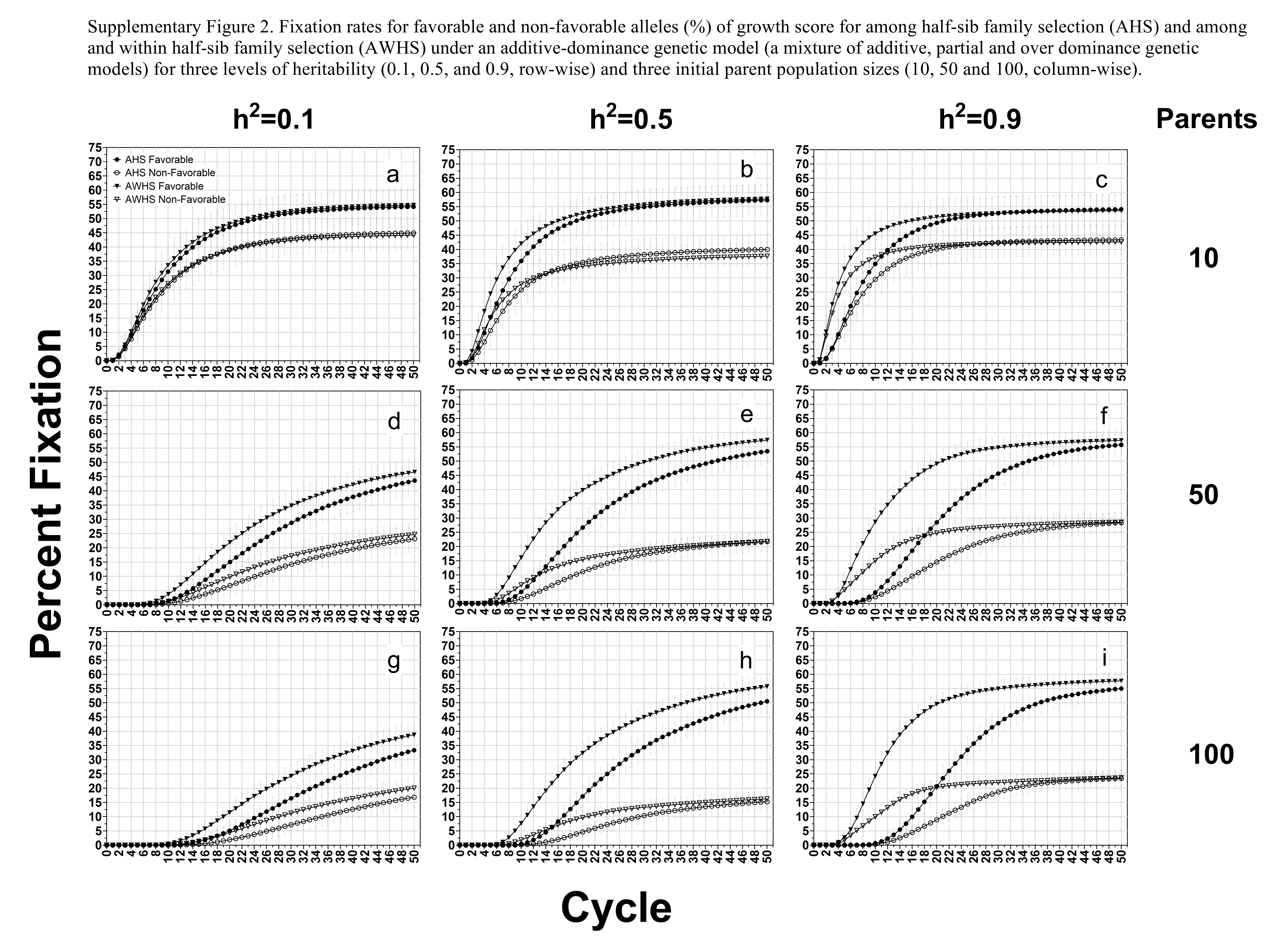

Supplement: Supplementary file 3 — Supplementary Figure 2 [file 41437_2018_156_MOESM3_ESM.tif]

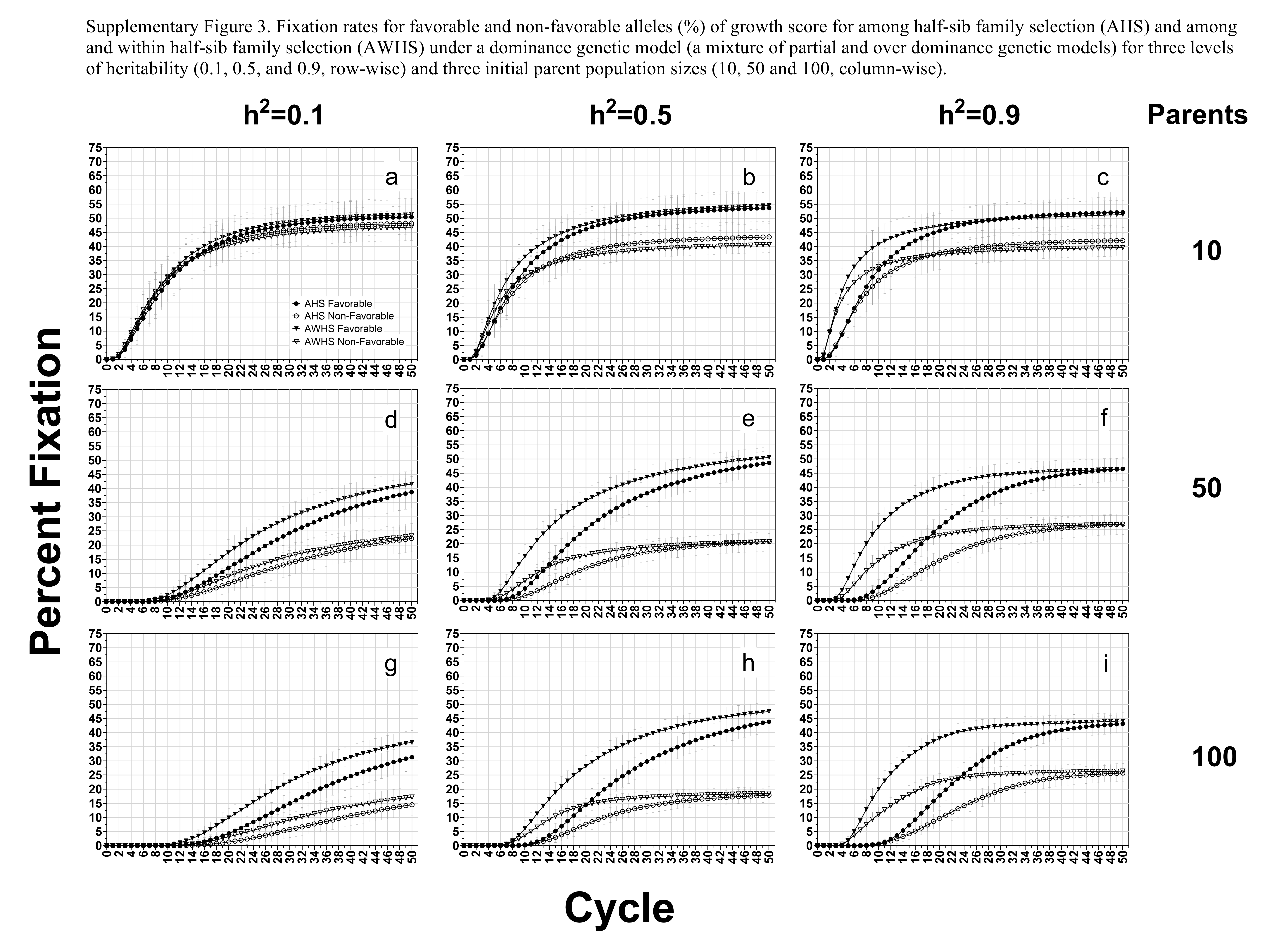

Supplement: Supplementary file 4 — Supplementary Figure 3 [file 41437_2018_156_MOESM4_ESM.tif]
